# Some Notes on Styracol

**Published:** 1907-03

**Authors:** Charles Reinhardt


### SOME NOTES ON STYRACOL.

BY

CHARLES REINHARDT, M.D.

FOR several years after having established the Hailey Open-air Sanatorium for consumption on the Chiltern Hills in Oxfordshire, it was my principle to avoid the administration of drugs so far as possible, and, indeed, medicines were seldom employed excepting for special emergencies.

Nothing has happened to convince me that in ordinary cases of phthisis medicinal treatment in the form of drugs administered internally is of any pronounced advantage amongst patients undergoing the open-air treatment in a sanatorium. I am even inclined to the opinion that those who suffer from incidental troubles, such as dyspepsia, diarrhœa, or cough, are as a rule likely to do quite as well if drugs are withheld, excepting in unusual cases. There is, however, one medicament of which I was forced to make an exception on account of the benefit derived by patients from its use, and that is guaiacol, which in my opinion is one of the most useful of drugs in the treatment of phthisis.

I was first obliged to arrive at this conclusion owing to an involuntary experiment. A patient—W. D.—arrived at the institution suffering from extensive cavitation of the left lung, both lobes of which were involved, the right lung being apparently free. The case was a chronic one, and had been under treatment at several other sanatoria. There was a moderate amount of cough and expectoration, but the temperature was normal, and I was able to give a fairly good prognosis. The patient had been taking guaiacol regularly, but on my advice this was at once discontinued.

It often happens that patients entering an open-air sanatorium make rapid and immediate progress, the symptoms abating and an increase of comfort and of vigour being experienced. This reaction is, of course, gratifying, even though the rate of progress is not always maintained. In the case to which reference is being made, however, the reaction or early access of improvement did not occur, to the disappointment both of the patient and myself, and for some time the condition of the former remained almost stationary, though there was a slight increase in the amount of cough, and an undoubted increase in the râles at the apex of the right lung. There was also some amount of diarrhœa. I attributed the want of apparent benefit partly to the chronicity of the case and the fact that the patient was in no sense new to sanatorium life, and partly to a certain boisterousness and excitability of temperament which he displayed. He, however, insisted that his progress had ceased when he gave up his diurnal doses of guaiacol. After six weeks had elapsed without noticeable benefit. a sudden improvement commenced, and it went on without interruption. Some time afterwards I found him in the act of taking a dose of guaiacol, and to my surprise he told me that he had recommenced the regular use of this drug six weeks after admission, which corresponded to the date when the improvement commenced.

I then sanctioned the use of the guaiacol, or rather replaced it by styracol, which I knew to be a combination of guaiacol and cinnamic acid, free from some of the disadvantages of guaiacol, and I was pleased to find that the patient continued to make progress as long as he remained in my sanatorium. He never again suffered from diarrhœa, but he eventually made a good recovery, is now married and in good health.

This case led me to employ guaiacol, chiefly in the form of styracol, in a number of instances, almost always with encouraging results ; but owing to the unpleasantness of the creosote-like flavour of guaiacol and to the tastelessness of styracol, and the fact that the latter only splits up into its components, guaiacol and cinnamic acid, when it has passed through the pylorus, I soon came to favour styracol, and to discard the use of guaiacol.

In all cases in which I suspected intestinal tuberculosis, or in which there was anything more than accidental diarrhœa, I administered styracol, and nearly always with advantage.

I also employed it in cases in which there was much cough, expectoration, or moisture in the lung, and I generally found these conditions were improved by its use.

In one instance, a patient suffering from a very large cavity occupying a considerable portion of the upper lobe of the right lung, was troubled with an extremely offensive expectoration, and a fœtor of breath which I was quite unable to assuage. The administration of styracol, however, eventually succeeded in reducing these unpleasant conditions to a remarkable extent, and if the dosage was omitted for any length of time they soon returned, though not quite to the same extent as before.

I have never found any ill effects follow the exhibition of styracol, and I have found benefits which were quite in excess of those following the administration of guaiacol in similar cases. This may be attributable to the therapeutic effects of the cinnamic acid, which is one of the components of styracol.

I have employed styracol in the form of powder and that of tablets. In the latter case I always recommended patients to bite them into minute particles, in order to secure that they should not pass through the alimentary system unchanged. The act of masticating styracol is not unpleasant, as the tablets are practically tasteless.

In my opinion styracol is one of the best available intestinal antiseptics, and its continual exhibition seems to impregnate the organism with guaiacol, so that the beneficial influence of that drug upon lung tissue undergoing necrosis is exerted to the full.

